# Utility of Cardiopulmonary Exercise Testing in Assessing Beta‐Blocker Efficacy in LQTS: Moving Away From One‐Size‐Fits‐All

**DOI:** 10.1111/jce.70001

**Published:** 2025-07-14

**Authors:** Iqbal El Assaad, Alison K. Heilbronner, Kenneth Zahka, Benjamin Hammond, Akash Patel, Peter F. Aziz

**Affiliations:** ^1^ Children's Institute Department of Heart, Vascular & Thoracic, Division of Cardiology & Cardiovascular Medicine Cleveland Clinic Children's Cleveland Ohio USA; ^2^ Cleveland Clinic Lerner College of Medicine Cleveland Ohio USA; ^3^ Department of Pediatric Cardiology Primary Children's Hospital Salt Lake Utah USA

**Keywords:** cardiopulmonary exercise testing, long QT syndrome, nadolol

## Abstract

**Background:**

Currently, there is no specific standard to assess beta blocker efficacy in long QT syndrome (LQTS).

**Objective:**

To describe our institutional experience with utilizing cardiopulmonary exercise testing (CPET) to assess for chronotropic suppression and to compare frequency of life‐threatening events (LTEs) on intentional “submaximal” treatment to those on maximal treatment.

**Methods:**

We queried our Inherited Arrhythmia Registry and identified patients with LQTS who were on “submaximal” beta blocker doses (nadolol < 0.75‐mg/kg/day & propranolol < 2 mg/kg/day) with at least 6 months follow up. Adequate beta blockade effect was defined as at least 15% decrease from maximal HR.

**Results:**

The study included 127 LQTS patients: 47% on maximal therapy, 43% on submaximal therapy, and 10% not receiving treatment. Thirty three percent of patients were on submaximal therapy due to side effects, none in patients less than 10 years of age. Baseline characteristics were similar between the groups. There was no significant difference in LTEs between maximal and submaximal therapy (8% vs. 5.4%, *p* = 0.72). During CPET, patients on maximal therapy were more likely to exhibit adequate chronotropic suppression (60% vs. 40%, *p* = 0.01). None of the patients on submaximal therapy with adequate chronotropic effect experienced LTEs during follow‐up.

**Conclusions:**

Adequate chronotropic suppression was achieved with “submaximal” beta blocker dose in 40%. Despite similar baseline risk profiles, LTEs were not significantly different in patients with submaximal versus maximal therapy. CPET may be a useful modality to devise an individualized treatment plan, especially in those who cannot tolerate the recommended guideline directed dose.

**Abbreviations**
CPET: cardiopulmonary exercise testing
ICDs: Implantable Cardiac Defibrillators.
LQTS: Long QT Syndrome
LTE: life threatening events

## 1 Introduction

Long QT syndrome (LQTS) is an inherited ion channelopathy characterized by prolonged QTc and abnormal T wave morphology on electrocardiogram (ECG) and can lead to life‐threating ventricular arrhythmias [1, 2, 3]. Current guidelines recommend nonselective beta blocker treatment as first‐line therapy in all patients with LQTS [2, 3, 4]. Experts in the field advise using nadolol at 0.75–2 mg/kg/day or propranolol at 2–3 mg/kg/day [4, 5, 6, 7], but there are no available studies defining the most effective dose, and there is variability in patient response to different doses [8, 9].

The diagnostic utility of exercise stress testing (EST) in patients being evaluated for LQTS is well established [10, 11, 12]. However, the utility of EST and more specifically CPET in assessing medication efficacy and need for therapy escalation is not well studied. We have observed that some patients experience significant side effects or cannot tolerate the full recommended dose of beta blockers. One study reported that 44% of patients treated with beta blockers experience side effects, the most common being fatigue, lightheadedness, and weight gain [9]. Side effects are also a common reason for patients to be noncompliant with their prescribed dose [6]. To address this challenge, we have developed an approach using CPET to assess the effectiveness of beta blocker therapy by evaluating chronotropic suppression during maximal exercise. CPET is the gold standard to determine aerobic fitness, and it provides the ability to examine integrated physiological responses during exercise [13]. Unlike routine EST, using the respiratory exchange ratio (RER) with CPET allows us to ensure that the patient achieved maximal effort. This individualized approach has provided us with objective data to guide the management of these patients.

The primary objective of this study is to describe our institutional experience with beta blocker therapy in patients with LQTS, specifically focusing on the use of CPET to assess for chronotropic suppression and guide management in patients who cannot tolerate the full dose of beta blockers. The secondary aim is to compare the frequency of life‐threatening events (LTEs) between two groups: those on intentional submaximal beta blocker dose and those on the full dose. Our hypothesis is that adequate chronotropic suppression can be achieved with submaximal beta blocker dosing, and CPET can be a useful tool to titrate the beta blocker dose to achieve the desired therapeutic effect in patients who cannot tolerate the recommended full dose. We also hypothesize that intentional submaximal dosing, guided by physicians, is not associated with an increased frequency of LTEs in certain patients.

## 2 Methods

We conducted a retrospective cohort study at Cleveland Clinic Children's to investigate the treatment and outcomes of patients with LQTS between January 2012 and 2023. At our institution, we have an Inherited Arrhythmia (IA) Clinic in which we evaluate patients of all ages with various inherited channelopathies, including LQTS. The study included both patients with known gene variants and those with elusive gene results. Patients with acquired long QT syndrome and those with less than 6 months of follow‐up were excluded from the analysis. The study protocol was approved by the Institutional Review Board at Cleveland Clinic Children's.

Data were collected from our internal Inherited Arrhythmia Database and electronic medical records. Data were stored and analyzed using a REDCap (Research Electronic Data Capture) database. Various variables were recorded, including patient demographics, gene test results, details of beta blocker therapy (type of beta blocker and dosage), QTc at the time of diagnosis and most recent visit, CPET variables (such as maximum heart rate, respiratory exchange ratio (RER), and QTc during recovery, and rhythm abnormalities), and the occurrence of life‐threatening events (LTEs), including cardiac syncope, appropriate implantable cardioverter‐defibrillator (ICD) shocks, sudden cardiac arrest, or sudden cardiac death.

Submaximal beta blocker therapy was defined as a dose of < 0.75 mg/kg/day for nadolol and < 2 mg/kg/day for propranolol. Adequate beta blockade was determined as a reduction of at least 15%–20% from the predicted maximal heart rate on steady‐state beta blocker dosing [4]. Only metabolic exercise stress tests with an RER ≥ 1.1 were included in the CPET analysis to ensure that the chronotropic suppression observed was a result of the beta blocker's effect and not due to suboptimal effort during the test. Our center utilizes a ramp protocol with time points and workload corresponding to a standard Bruce protocol (e.g., stage 2 begins at the 3‐min mark with a 12% grade and 2.5 mph and then gradually ramps up to achieve stage 3 by the 6‐min mark).

Continuous variables were expressed as mean ± standard deviation (SD), while categorical variables were presented as counts and percentages. To compare the demographic and clinical characteristics between study groups, Wilcoxon rank sum or two‐sample *t*‐tests were used for continuous or ordinal variables, and Chi‐square or Fisher's exact tests were used for categorical variables. The comparison of event rates between the two groups was performed using the Chi‐square or Fisher's exact test. Total duration on maximal and submaximal therapy were recorded and event rates per 100 patient‐years were calculated. All statistical tests were two‐tailed, and a significance level of 0.05 was used to determine statistical significance.

## 3 Results

### 3.1 Study Subjects

**Figure 1:**
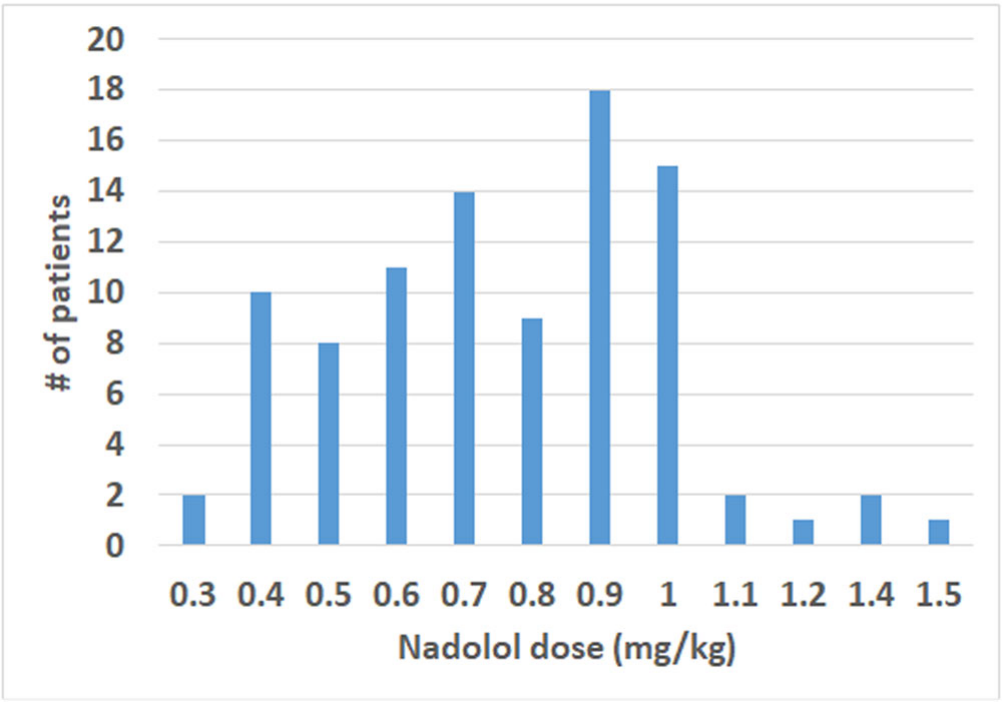
Distribution of nadolol dose among patients (*n* = 93).

Of the total 211 patients in our Inherited Arrhythmia Database, 127 patients with long QT syndrome (LQTS) met our inclusion criteria and were included in the analysis. Table 1 depicts baseline characteristics of the studied cohort. The mean age at diagnosis was 12.7 ± 11.9 years, and the mean corrected QT (QTc) was 475 ± 36 ms. Most of the patients were female, accounting for 57% of the cohort. The most prevalent genotype was LQTS type 1 (LQT1), which was observed in 43% of the patients. A total of 14 patients (11%) had implantable cardioverter‐defibrillators (ICDs). The majority (115/127, 91%) received beta blocker therapy, with nadolol being the most prescribed beta blocker, given to 93 out of 127 patients (73%). Distribution of nadolol dosage in milligrams per kilogram (mg/kg) among the patients is illustrated in Figure 1.

**Table 1:** Baseline demographics comparing characteristics between patients on maximal therapy versus those on submaximal therapy.

	Total (*n* = 127)	Maximal Therapy (*N* = 60)	Submaximal Therapy (*N* = 55)	None (*n* = 12)	*p* value*
Mean age at diagnosis, ± SD	12.7 ± 11.9	8.4 ± 8.3	15 ± 12	21 ± 16	< 0.001
Median age at diagnosis, (IQR 25th−75th)	11.1 (1.6–17.5)	8.6 (0.35–13.6)	13.3 (7–20)	18 (5–33)	
Female, *n* (%)	72 (57)	35 (58)	28 (50)	9 (75)	0.42
Medication, *n* (%)					
Nadolol	93 (73)	46 (77)	47 (85)	0	0.23
Propranolol	22 (17)	14 (23)	8 (15)	0	
None	12 (10)	0	0	12 (100)	
LQTS type, *n* (%)					
1	55 (43)	30 (50)	20 (36)	6 (50)	0.28
2	44 (35)	19 (32)	20 (36)	4 (34)	
3	9 (7)	2 (3)	6 (11)	1 (8)	
Others	19 (15)	9 (15)	9 (16)	1 (8)	
Mean QTc at time of diagnosis, ± SD	475 ± 36	472 ± 29	480 ± 42	465 ± 41	0.25
Median QTc at time of diagnosis, (IQR 25th–75th)	475 (450–500)	470 (451‐495)	472 (454‐503)	462 (432‐485)	
Mean QTc at most recent visit, ± SD	464 ± 36	458 ± 33	468 ± 32	455 ± 27	0.04
Median QTc at most recent visit, (IQR 25th–75th)	458 (441–483)	452 (437–479)	467 (447–489)	448 (434–471)	
QTc > 500 at diagnosis, *n* (%)					
Yes	30 (24)	11 (18)	18 (333)	1 (8)	0.89
No	86 (68)	44 (73)	34 (61)	8 (67)	
QTc > 500 at last f/u, *n* (%)					
Yes	18 (14)	7 (12)	10 (18)	1 (8)	0.36
No	107 (84)	51 (85)	45 (82)	11 (92)	
Mean f/u time, ± SD	7.7 ± 6.78	7 ± 4.96	8.6 ± 7.9	6.9 ± 8.9	0.18
Median f/u time, (IQR 25th–75th)	5.96 (2.96–10)	5.6 (3.5–8.3)	6.8 (2.5–13.3)	2.5 (1.2–12)	
Reason for referral, *n* (%)					
QTc prolongation	26 (20)	15 (25)	9 (16)	2 (17)	0.53
ACA	5 (4)	1 (2)	4 (7)	0	
Cardiac syncope	18 (14)	8 (13)	8 (15)	2 (17)	
FH	74 (58)	34 (57)	32 (58)	8 (66)	
Other	4 (3)	2 (3)	2 (4)	0	
Reason for referral, *n* (%)					
ACA/Cardiac syncope	23 (18)	9 (15)	12 (22)	2 (17)	0.34
Age at diagnosis > 30, *n* (%)					
Yes	12 (9)	0 60 (100)	8 (15)	4 (33)	0.0021
No	115 (91)		47 (85)	8 (67)	
Age at last f/u > 30, *n* (%)					
Yes	27 (21)	4 (7)	16 (29)	7 (58)	0.0025
No	100 (79)	56 (93)	39 (71)	5 (42)	
Wt > 100 kg					
Yes	12 (9)	2 (3)	9 (16)	1 (8)	0.025
No	115 (91)	58 (97)	46 (83)	11 (92)	
Mean weight at last f/u, ± SD	62 ± 29	50 ± 25	74 ± 27	66 ± 34	< 0.001
Events before beta blocker, *n* (%)	27 (21)	9 (15)	16 (29)	2 (16)	0.07
Events on beta blocker, *n* (%)	8 (6.2%)	5 (8%)	3 (5.4%)	0	0.72
Side effects, *n* (%)					
Yes	29 (23)	6 (10)	18 (32)	5 (42)	0.003
No	98 (77)	54 (90)	37 (67)	7 (58)	
Devices, *n*		10	7	5	
ICD	14	5	4	4	
PPM	4	2	1	0	
Loop recorder	6	3	2	1	
ICD/PPM, *n* (%)					
Yes	18 (14)	7 (12)	5 (10)	4 (33)	0.76
No	109 (86)	53 (88)	50 (90)	8 (67)	

Abbreviations: IQR, interquartile range; ACA, aborted cardiac arrest; FH, family history; ICD, implantable cardiac defibrillator; PPM, permanent pacemaker.

* *p* value represents comparison between patients on maximal versus submaximal therapy.

### 3.2 β‐Blocker Dosage

Out of the 127 patients, 60 (47%) were receiving maximal therapy, 55 (43%) were on submaximal therapy, and 12 (10%) were not receiving any medical treatment. A comparison between patients receiving maximal therapy and those on submaximal therapy revealed that patients on maximal therapy were younger at the time of diagnosis (8.4 ± 8.3 years vs. 15 ± 12 years, *p* = 0.0003). There were no significant differences in the mean QTc duration at the time of diagnosis between the two groups (475 ± 36 ms for maximal therapy vs. 472 ± 29 ms for submaximal therapy). The genotype profile distribution between the groups was also similar (*p* = 0.28). Furthermore, there were no significant differences in the history of cardiac syncope or aborted cardiac arrest between patients on maximal therapy and those on submaximal therapy (15% vs. 22%, *p* = 0.34). The percentage of patients with pacemakers or implantable cardiac defibrillators was also similar between the groups (12% for maximal therapy vs. 10% for submaximal therapy, *p* = 0.76). Regarding life threatening events on beta blocker therapy, there was no statistically significant difference in the occurrence of such events between the patients on maximal therapy and those on submaximal therapy (8% vs. 5.4%, *p* = 0.72).

### 3.3 Cardiopulmonary Exercise Testing

**Figure 2:**
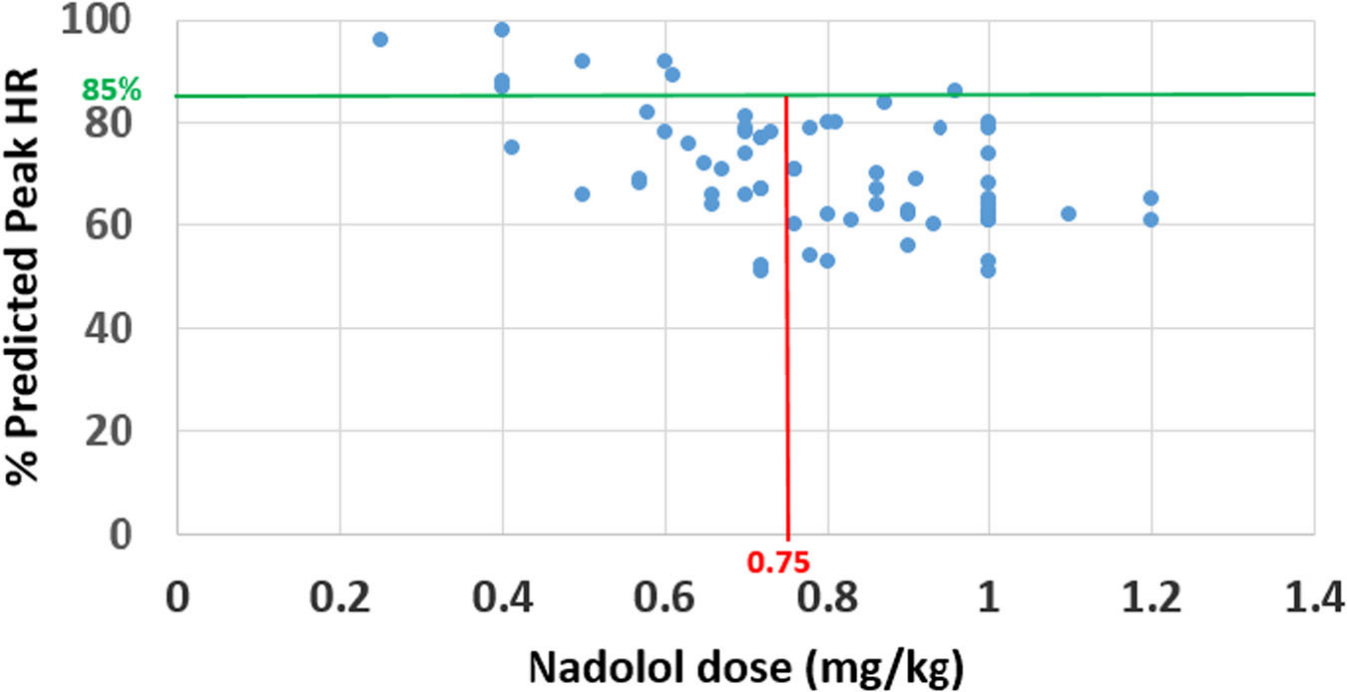
Predicted heart rate (HR) as a function of nadolol dose (*n* = 66).

Out of the total 127 patients included in the study, 74 patients (58%) underwent at least one CPET. Sixty‐nine CPETs in 49 patients were performed on beta blocker therapy and had an RER > 1.1 signifying that this was a maximal effort test. Of those, 66 were performed on nadolol therapy and three were performed on propranolol therapy. In patients whom were treated with nadolol, 36 (55%) were on maximal therapy versus 30 (45%) on submaximal therapy. Of the 30 patients on submaximal therapy, 12 (40%) achieved adequate chronotropic suppression, and none of them had a LTE during follow up. Patients on maximal therapy were more likely to exhibit adequate chronotropic suppression compared to patients on submaximal therapy (60% vs. 40%, *p* = 0.01; Figure 2).

Of the 49 patients with CPET and RER > 1.1, only 13 patients (26%) had baseline testing before therapy initiation. Of those, only six patients had a metabolic EST. Four out of the six achieved a normal maximal heart rate and two had a blunted maximal heart rate (defined as predicted HR < 85%).

### 3.4 Events on Beta Blocker Therapy

During the mean follow‐up period of 7.7 years, a total of eight patients (6.2%) experienced cardiac events after their initial diagnosis. While receiving beta blocker therapy, the overall event rate was 1.1 per 100 patient‐years. In the submaximal group, three events occurred over 203.7 years of follow up, corresponding to an event rate of 1.5 per 100 patient‐years. In contrast, the maximal group experienced five events over a total of 506.2 years of follow up, yielding an event rate of 1.0 per 100 patient‐years. The incidence rate between the two groups was similar, with an incidence rate ratio of 1.5 (95% CI 0.2–7.7, *p* = 0.59).

### 3.5 Events on Submaximal Therapy

The first patient on submaximal therapy was a 57‐year‐old male who was diagnosed with LQTS type 2 at the age of 51 through family cascade screening. Despite having no history of LQTS cardiac events, his baseline ECG showed prolonged QTc at 540 ms and abnormal T‐wave morphology. He was initiated on nadolol therapy at a dose of 40 mg (0.55 mg/kg). However, at the age of 57, he experienced an out‐of‐hospital cardiac arrest with no identifiable triggers.

The second patient was a 35‐year‐old female who was diagnosed with LQTS type 2 following a seizure‐like episode in the postpartum period at the age of 20. She initially started beta blocker therapy but discontinued it due to medication side effects. Despite consistently having a QTc > 500 ms and multiple discussions, she refused beta blocker therapy. As a result, she underwent primary prevention transvenous ICD placement. She experienced repeated appropriate shocks for ventricular fibrillation (VF) and could only tolerate a low dose of nadolol at 20 mg (0.25 mg/kg). An EST on submaximal therapy showed inadequate chronotropic suppression and despite our recommendations for increased beta blocker dose, the patient declined. Subsequently, she underwent left cardiac sympathetic denervation and is currently maintained on nadolol 20 mg daily (0.25 mg/kg) without recurrent shocks since her sympathectomy.

The third patient was a 23‐year‐old female with LQTS type 1, carrying two deleterious variants on the LQT1 gene. She was diagnosed at the age of 4 after experiencing a syncopal event while swimming. Her baseline QTc consistently exceeded 550 ms. At the age of 8, she had a breakthrough syncopal event in the setting of outgrowing her nadolol dose since her last visit (0.7 mg/kg).

### 3.6 Events on Maximal Therapy

The fourth patient was diagnosed with LQTS types 1 and 2 during the newborn period through family cascade screening. The baseline ECG showed a QTc of 450 ms, and the patient was treated with propranolol at a dose of approximately 3 mg/kg/day. At age 3, the patient experienced a seizure‐like event at home, which raised concerns of bradycardia‐induced Torsades de Pointes in addition to hypoglycemia as the latter would not explain the elevated troponin levels. As a result, the patient underwent epicardial pacemaker placement and has remained free from cardiac events for the past 12 years.

The fifth patient initially had a misdiagnosis of seizures for the first 3 years of life and at age 4 experienced exertional syncope, which prompted a referral to cardiology. The correct diagnosis of Jervell and Lange‐Nielsen syndrome was made, and the patient had a baseline QTc of 518 ms. Treatment with nadolol at a dose of 1 mg/kg was initiated, but the patient had breakthrough syncope while on therapy, leading to ICD placement at age 6. The patient is currently doing well and has not required any ICD therapies.

The sixth patient was diagnosed with LQTS type 2 shortly after birth due to the presence of Torsades de Pointes and pseudo‐heart block. The patient underwent epicardial pacemaker placement soon after birth and has been maintained on nadolol therapy at a dose of 1 mg/kg. The QTc has consistently been > 500 ms. At the age of 9, the patient experienced a single self‐resolved episode of Torsades de Pointes during ocular surgery, with the ventricular pacing rate adjusted to 40 bpm. Since then, the VVI rate has been increased to 60 bpm, and the patient has remained asymptomatic.

The seventh patient was diagnosed with LQTS type 1 at birth through familial cascade screening. The baseline QTc was 547 ms. At the age of 11, the patient experienced a cardiac arrest while on nadolol therapy at a dose of 1.2 mg/kg, leading to the placement of an ICD for secondary prevention. The patient has not received any ICD therapies since then.

The eighth patient, a 19‐year‐old female, was diagnosed with LQTS type 2 at the age of 12 following exertional syncope. Due to significantly prolonged baseline QTc of 540 ms and a history of noncompliance, the patient underwent ICD placement for primary prevention. However, despite being on maximal nadolol therapy at a dose of 1.35 mg/kg, the patient experienced two additional appropriate shocks for ventricular fibrillation.

### 3.7 Reasons for Submaximal Therapy

**Figure 3:**
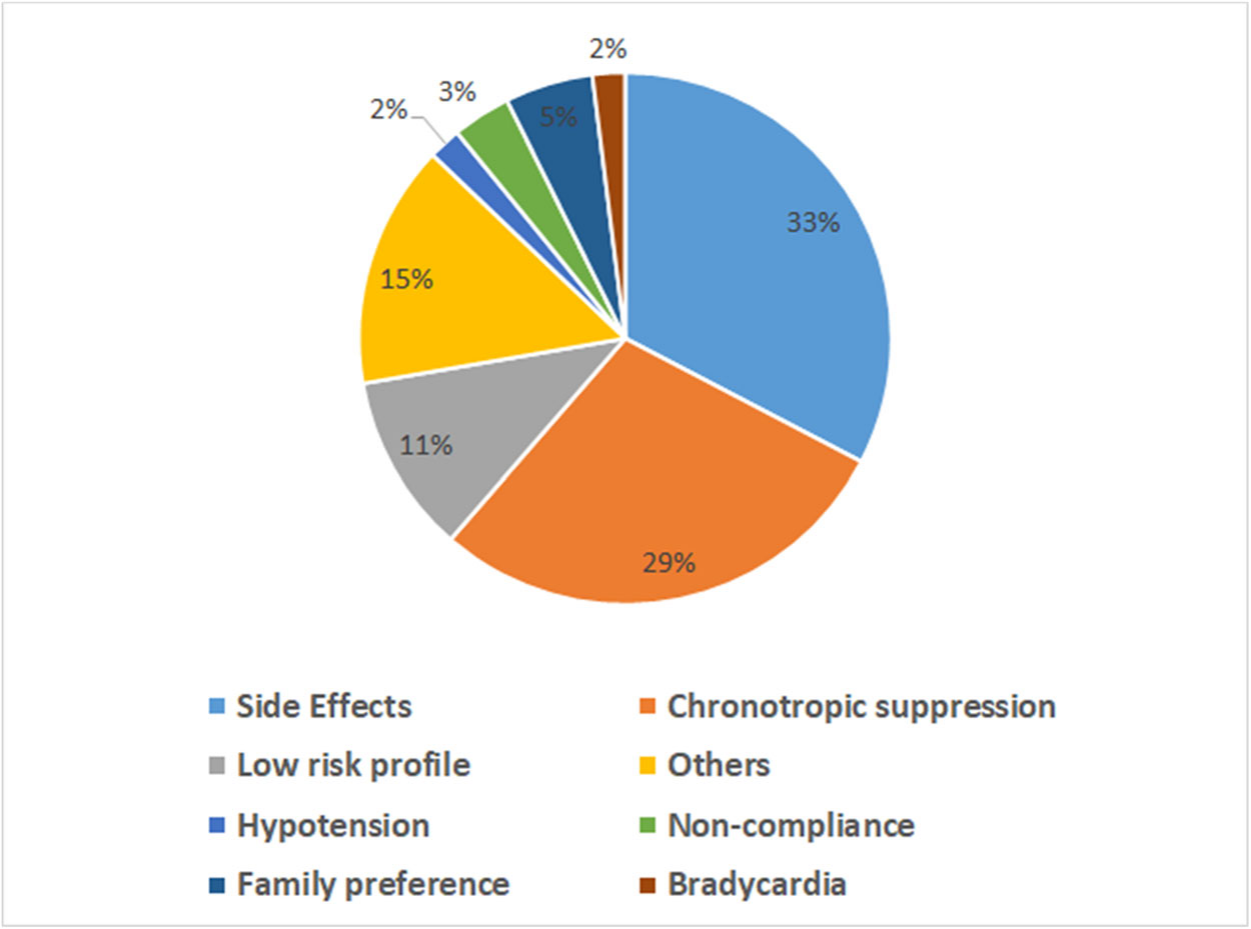
Reasons for submaximal therapy (*n* = 55).

Among the reasons for submaximal therapy, the most common factor was the occurrence of side effects, reported by 18 patients (33%). Other reasons included achieving adequate chronotropic suppression (16, 29%), low risk profile in genotype positive phenotype negative patients (6, 11%), family preference (3, 5.4%), noncompliance (2, 3.6%), bradycardia (1, 2%), low blood pressure (1, 2%), and other miscellaneous reasons (8, 15%) (Figure 3). The reported side effects included fatigue in 14, depression in 5, anxiety in 1, dizziness in 5, and weight gain in 2. Side effects were found to be more likely to occur in patients diagnosed with LQTS at the age of 10 years or older (50% vs. 26%, *p* = 0.025). It is worth noting that at the time of the last follow‐up, only two patients under the age of 10 were intentionally not started on beta blocker medications due to their low risk profile.

## 4 Discussion

This retrospective cohort study aimed to describe our institutional experience, management practices, and outcomes of patients with LQTS, with a focus on the role of CPET in guiding management. Our study highlights the following four major points: (1) a substantial number of patients can achieve adequate chronotropic suppression on submaximal beta blocker dose; (2) risk of LTEs in patients on submaximal beta blocker therapy is similar to those on maximal therapy; (3) many patients experience significant side effects secondary to beta blocker therapy and cannot tolerate maximal doses supporting the need for personalized treatment plan; (4) some patients are still at risk of LTE regardless of being on maximal beta blocker therapy and this is due to their high risk profile (Central Illustration 1).

**Central Illustration 1:**
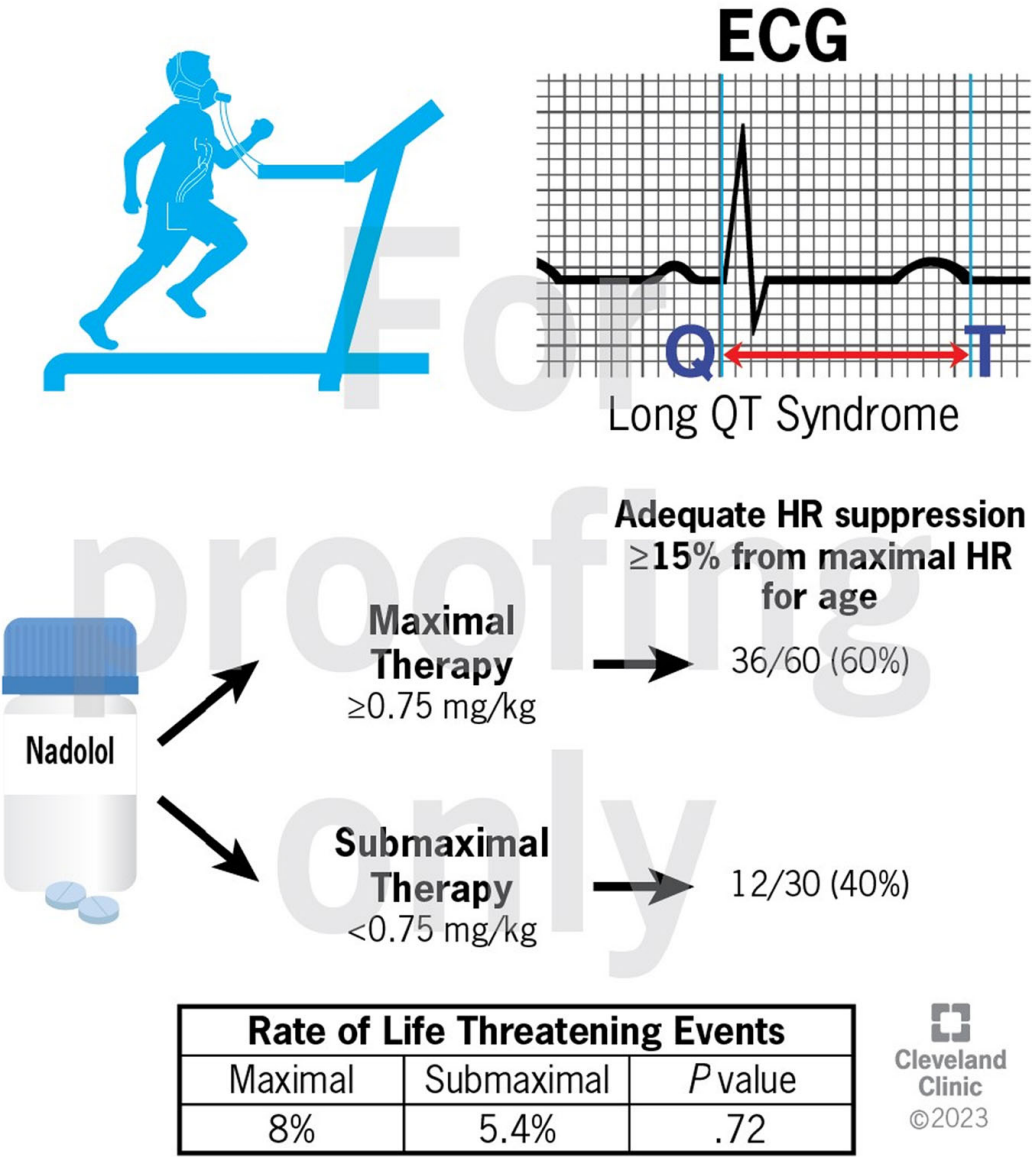
Utility of cardiopulmonary exercise testing in assessing beta blocker efficacy in LQTS: moving away from One‐Size‐Fits‐All.

Our study investigated the effects of submaximal dosing of beta blockers on achieving chronotropic suppression and its impact on the rates of LTEs. Our data revealed that 40% of LQTS patients achieved adequate chronotropic suppression despite receiving submaximal treatment, and none of these patients experienced LTEs during the follow‐up period. These results are consistent with previous studies that have observed variability in the dose response to beta blockers [8]. For instance, another study examining nadolol usage found no correlation between the prescribed dose and maximal heart rate during EST. The study in question also reported similar findings, with 35% of their cohort requiring less than the median dose for adequate suppression. In contrast, our study demonstrated that 77% of patients on submaximal therapy achieved adequate chronotropic suppression. The variation in the proportion of patients achieving adequate blunting on submaximal therapy between the two studies may be attributed to differences in the definition of submaximal therapy, definition of adequate heart rate suppression (70% of the age predicted maximum heart rate was used in this study as a target) or other unprovided demographic differences among patients. Additionally, a major strength of our study is that we utilized CPET instead of routine EST, which provides a reliable evaluation of the patient's physiological response to maximal effort [13]. We were able to ensure that the observed chronotropic suppression is a true representation of the patient's maximal effort rather than a consequence of submaximal effort.

Side effects while on beta blockers are a significant obstacle in providing care. One previous study demonstrated that 44% of patients on beta blocker treatment for LQTS experienced side effects. The most common side effect in patients receiving beta blockers was fatigue, which was reported in 35% of their entire cohort [9]. Furthermore, the negative impact on quality of life has been reported to lead to 8‐10% of patients receiving beta blocker therapy to discontinue their treatment [6, 9]. The data in our study suggests that patients receiving submaximal therapy often exhibit adequate chronotropic suppression by CPET. Therefore, perhaps it would still be safe to prescribe lower dosages of beta blockers to alleviate the burden of side effects in certain patients, which would hold the potential to significantly improve the quality of life for many patients. It is important to note that submaximal dosing should not be used as a universal approach, as there are still patients who experience breakthrough events despite being on submaximal therapy. Therefore, it is crucial that this approach is implemented intentionally and under the guidance of an experienced inherited arrhythmia specialist after careful risk stratification of the patient.

Among our cohort, 33% of patients reported side effects from treatment with a beta blocker; a finding that is concordant with previous studies [2, 9]. Importantly though, our results suggest that younger patients (< 10 years) tolerate higher doses than older patients, because none of the participants in this age group reported any medication side effects. We believe that younger patients tolerate beta blockers better than older patients because they are initiated on therapy early in life and therefore have no baseline experience or a reference point for how they feel without the medication. Additionally, there were no differences in event rate in the patients on maximal therapy (relatively younger at the time of diagnosis) versus those on submaximal therapy (relatively older at the time of diagnosis). This altogether emphasizes the importance of utilizing CPETs to direct treatment in patients with LQTS. The results from our study suggest that by completing an CPET around the age of 10 years and before puberty, this could better identify patients who might have adequate chronotropic suppression on a submaximal dosage. This approach, guided by the CPET results, could potentially reduce the incidence of side effects in this patient population compared to simply adjusting the dosage based on weight without correlating it with CPET findings. However, further studies are necessary to explore and validate this recommendation.

Furthermore, it has been observed in other studies that despite receiving treatment, breakthrough cardiac events can still occur in patients with LQTS. For instance, one study reported a 3% incidence of arrhythmic events (cardiac syncope and ventricular tachycardia/ventricular fibrillation) [8]. In our study, the event rate was 6.2%, with five documented arrhythmias requiring intervention and two cases of cardiac syncope. The differences in event rates between the studies could potentially be explained by variations in the proportions of LQTS types/genotypes. The previous study specifically presented data on patients with LQTS1, LQTS2, or mixed phenotypes. In contrast, our study included a broader range of LQTS genotypes, which could suggest a higher proportion of patients with more malignant phenotypes. Additionally, our study had a greater percentage of patients with implantable cardioverter‐defibrillators (ICDs), which could also indicate that our patient population overall had a higher prevalence of severe forms of LQTS.

## 5 Limitations

Our study is subject to several limitations that should be taken into consideration when interpreting the results. Firstly, the generalizability of the findings may be limited as the data was collected from a single center. Secondly, although we utilized our center's internal Inherited Arrhythmia Database, which has been actively collecting data since 2012, the sample size was relatively small. Additionally, among the 127 patients included in the study, only 66 met the inclusion criteria for CPETs with RER > 1.1, potentially introducing selection bias. Also, we did not consider medication compliance in this study, which could have provided further information pertaining to usage of beta blockers and chronotropic suppression along with cardiac events. Lastly, we assume that the protective role of beta blockers in patients with LQTS is specifically related to heart rate suppression. Although the mechanism is incompletely understood, it is thought that beta blockers reduce the rate of cardiac events in LQTS patients due to attenuation of adrenergic‐mediated triggers [14, 15], which is why we considered this to be an adequate proxy measure. It has also been recently shown that some patients with long QT syndrome have primary chronotropic insufficiency at baseline [16]. The latter are two important limitations that are very relevant to this study and the reason why maximally tolerated therapy should be trialed in every patient. In our study, only a small percentage of patients who underwent CPET following therapy initiation—and demonstrated an RER > 1.1—had undergone baseline CPET. This is primarily attributable to a young age at the time of diagnosis or to a diagnosis established at another institution, among other factors. This represents a significant limitation and underscores the need for prospective data to accurately identify patients with baseline chronotropic insufficiency and compare their outcomes to those without. Considering these limitations and the findings from our study, it would be prudent for electrophysiologists to consider obtaining a baseline CPET to assess for chronotropic insufficiency as part of the diagnostic process. This recommendation is relevant when a blunted maximal heart rate is being utilized as an endpoint for beta blocker titration. While we continue to advocate for treatment with the maximally tolerated beta blocker dose, our suggested approach can be useful in patients who do not tolerate maximal medical therapy. This approach emphasizes our efforts to simultaneously balance patient safety, minimize side effects, and maximize the chances of medication compliance.

## 6 Conclusions

In our institutional experience with patients diagnosed with long QT syndrome (LQTS), 40% of patients achieved adequate chronotropic suppression with submaximal doses of beta blockers. Importantly, despite having similar baseline risk profiles, there was no significant difference in the occurrence of life‐threatening arrhythmias between patients on submaximal therapy and those on maximal therapy. These findings suggest that submaximal dosing of beta blockers may be effective in managing LQTS, and CPET can be a valuable tool for devising an individualized treatment plan and titrating the beta blocker dose to achieve the desired effect. This study highlights the potential benefit of tailoring beta blocker therapy based on individual patient response and tolerance, especially for those who cannot tolerate the recommended guideline‐directed dose. By utilizing CPET, clinicians can assess the extent of chronotropic suppression and adjust the dosage accordingly, providing personalized care to LQTS patients.

## Disclosure

The authors of this manuscript have no conflicts of interest relevant to this study to disclose.

## Data Availability

The data that support the findings of this study are available from the corresponding author upon reasonable request.
